# Decision on non-conveyance of patients suspected of COVID-19 in a novel arrangement with assessment visits by paramedics at home

**DOI:** 10.1186/s12873-023-00826-6

**Published:** 2023-05-26

**Authors:** Vibe Maria Laden Nielsen, Tim Alex Lindskou, Ulla Møller Weinreich, Michael Skærbæk Jespersen, Erika Frischknecht Christensen, Henrik Bøggild

**Affiliations:** 1 Centre for Prehospital and Emergency Research, Aalborg University Hospital, Aalborg, Denmark; 2 Department of Clinical Medicine, Aalborg University, Aalborg, Denmark; 3 Department of Respiratory Diseases, Aalborg University Hospital, Aalborg, Denmark; 4 Prehospital Emergency Services, North Denmark Region, Aalborg, Denmark; 5 Public Health and Epidemiology, Department of Health Science and Technology, Aalborg University, Aalborg, Denmark

**Keywords:** Coronavirus disease 2019, Non-conveyance, Emergency medical services, Paramedics

## Abstract

**Background:**

During the first weeks of the outbreak of the coronavirus disease 2019 (COVID-19), the North Denmark emergency medical services authorised paramedics to assess patients suspected of COVID-19 at home, and then decide if conveyance to a hospital was required. The aim of this study was to describe the cohort of patients who were assessed at home and their outcomes in terms of subsequent hospital visits and short-term mortality.

**Methods:**

This was a historical cohort study in the North Denmark Region with consecutive inclusion of patients suspected of COVID-19 who were referred to a paramedic’s assessment visit by their general practitioner or an out-of-hours general practitioner. The study was conducted from 16 March to 20 May 2020. The outcomes were the proportion of non-conveyed patients who subsequently visited a hospital within 72 hours of the paramedic’s assessment visit and mortality at 3, 7 and 30 days. Mortality was estimated using a Poisson regression model with robust variance estimation.

**Results:**

During the study period, 587 patients with a median age of 75 (IQR 59–84) years were referred to a paramedic’s assessment visit. Three of four patients (76.5%, 95% CI 72.8;79.9) were non-conveyed, and 13.1% (95% CI 10.2;16.6) of the non-conveyed patients were subsequently referred to a hospital within 72 hours of the paramedic’s assessment visit. Within 30 days from the paramedic’s assessment visit, mortality was 11.1% [95% CI 6.9;17.9] among patients directly conveyed to a hospital and 5.8% [95% CI 4.0;8.5] among non-conveyed patients. Medical record review revealed that deaths in the non-conveyed group had happened among patients with ‘do-not-resuscitate’ orders, palliative care plans, severe comorbidities, age ≥ 90 years or nursing home residents.

**Conclusions:**

The majority (87%) of the non-conveyed patients did not visit a hospital for the following three days after a paramedic’s assessment visit. The study implies that this newly established prehospital arrangement served as a kind of gatekeeper for the region’s hospitals in regard to patients suspected of COVID-19. The study also demonstrates that implementation of non-conveyance protocols should be accompanied by careful and regular evaluation to ensure patient safety.

**Supplementary Information:**

The online version contains supplementary material available at 10.1186/s12873-023-00826-6.

## Introduction

On 11 March 2020, the World Health Organization announced coronavirus disease 2019 (COVID-19) to be a pandemic [1]. During the first outbreak, Danish health authorities advised that patients suspected of COVID-19 without severe symptoms should neither be admitted to a hospital, nor show up at their general practitioner (GP) [2]. The advice was given in order to minimize the risk of transmitting the infection to others, as patients are most contagious in the 48 hours before clinical symptoms appear and early in the course of COVID-19 [3]. Before regular test centres or vaccine centres were operative, COVID-19 assessment clinics were heterogeneously organised across Denmark. Beginning on 16 March 2020, the North Denmark emergency medical services (EMS) established an arrangement in which paramedics assessed patients suspected of COVID-19 at home and decided if conveyance to hospital was required. Had the EMS not had this novel arrangement in place, the alternative for the GP would have been to summon an ambulance to bring the patient to hospital for a clinical assessment. The aim of this study was to describe the cohort of patients who were assessed by paramedics and released at home (non-conveyed) and their outcomes in terms of subsequent hospital visits and short-term mortality.

## Methods

### Study design and setting

This was a quality assurance project based on a historical cohort from the North Denmark Region with consecutive inclusion of patients suspected of COVID-19. The patients were referred to a paramedic’s assessment visit by their GP or an out-of-hours GP who had assessed their symptoms as suspected of COVID-19. Paramedics were dispatched via the regional Emergency Medical Coordination Centre. The study period is equal to the period in which this provisional arrangement was operating, from 16 March to 20 May 2020. Paramedics assessed patients at home using a standardised protocol that included assessment of vital signs [4], work of breathing, age, chronic comorbidities and optional point-of-care testing of C-reactive protein (Additional file 1. *COVID-19 instruction manual for paramedics*). Based on the evaluation, they decided to either perform a throat swab for a reverse transcription quantitative polymerase chain reaction (PCR) test and let the patient stay at home or to convey the patient to a COVID-19 assessment clinic. Physician advice was available via telephone, but not mandatory. The consulting physician could either be the referring GP, the on-call consultant in either infectious diseases or pulmonology or a prehospital physician specialised in anaesthesiology and intensive care medicine. The visiting paramedic decided on whom of those physicians to consult. No patient was released at home without a plan for whom to call in case of further progression of their disease.

### Data collection and outcomes

The study used routinely collected data from the logistic system, Logis CAD (*Logis Solutions, Nærum, Denmark*), which were linked with prehospital electronic patient medical records (ePMR). Hospital visits were retrieved via the Patient Administrative System, a bi-regional registry that covers the North Denmark and Central Denmark Region [5]. Data were pseudo-anonymised when linkage had been completed. Outcomes were the proportion of non-conveyed patients who subsequently visited a hospital within 72 hours of the paramedic’s assessment visit and mortality at 3, 7 and 30 days. Proportions are reported with 95% confidence intervals for the estimated population proportion. Co-morbidity was categorised into three groups using the Charlson Co-morbidity Index (CCI) [6]. If a unique patient had had more than one assessment visit during the study period, only the last visit was included in the mortality analyses. Incidence rates (crude IRs) of mortality were estimated using a “modified Poisson regression” model with robust variance estimation [7]. A Cox regression was performed with a dichotomised exposure (directly conveyed or non-conveyed patients). Assumptions for proportional hazards were assessed by a log-minus-log plot, Stata’s ‘estat phtest’ command and a Schoenfeld residual plot. Cases in which the patient died within 30 days of the visit were further investigated by review of the prehospital ePMR. We specifically searched for descriptions of’do-not-resuscitate’ orders, palliative care plans, severe comorbidities, or if the patient had either been aged ≥ 90 years or been a nursing home resident. Hospital diagnoses were reported using the International Classification of Diseases, Tenth Revision (ICD-10). The label ‘hospital diagnosis’ was used for the first chronological diagnosis the patient received during the hospital stay. If the label was a diagnosis from chapters XVIII (*Symptoms and abnormal findings, not elsewhere classified*) or XXI (*Factors influencing health status and contact with health services*), the successive organ-specific or cause-specific diagnosis was applied. Analyses were performed in Stata/MP 17.0 (*StataCorp LLC, TX 77845, USA*).

## Results

### Baseline characteristics

**Fig. 1:**
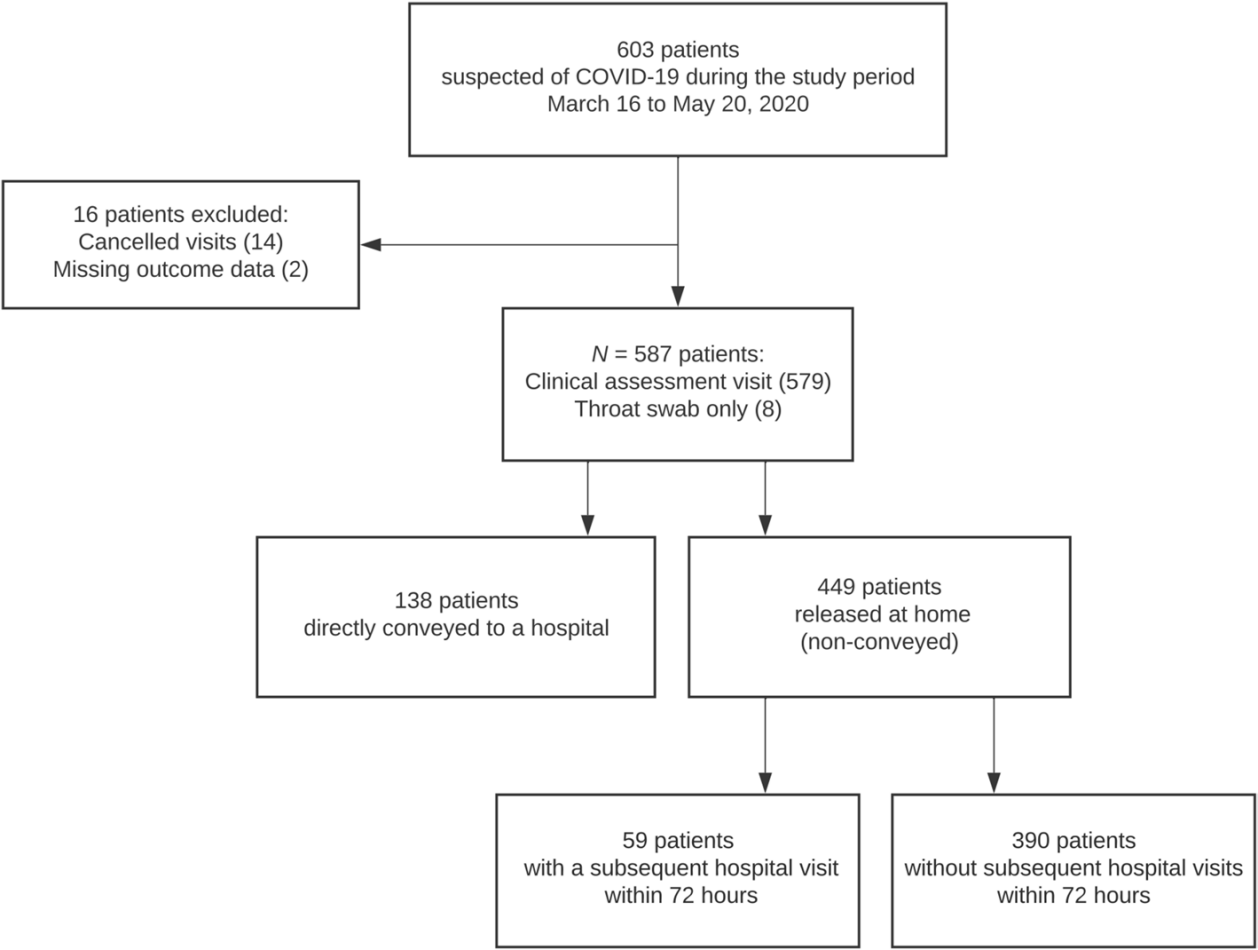
Flow diagram of the study population. A detailed outline of the patients who were referred by their GP or an out-of-hours GP to a paramedic’s assessment visit at home and how they proceeded

**Fig. 2:**
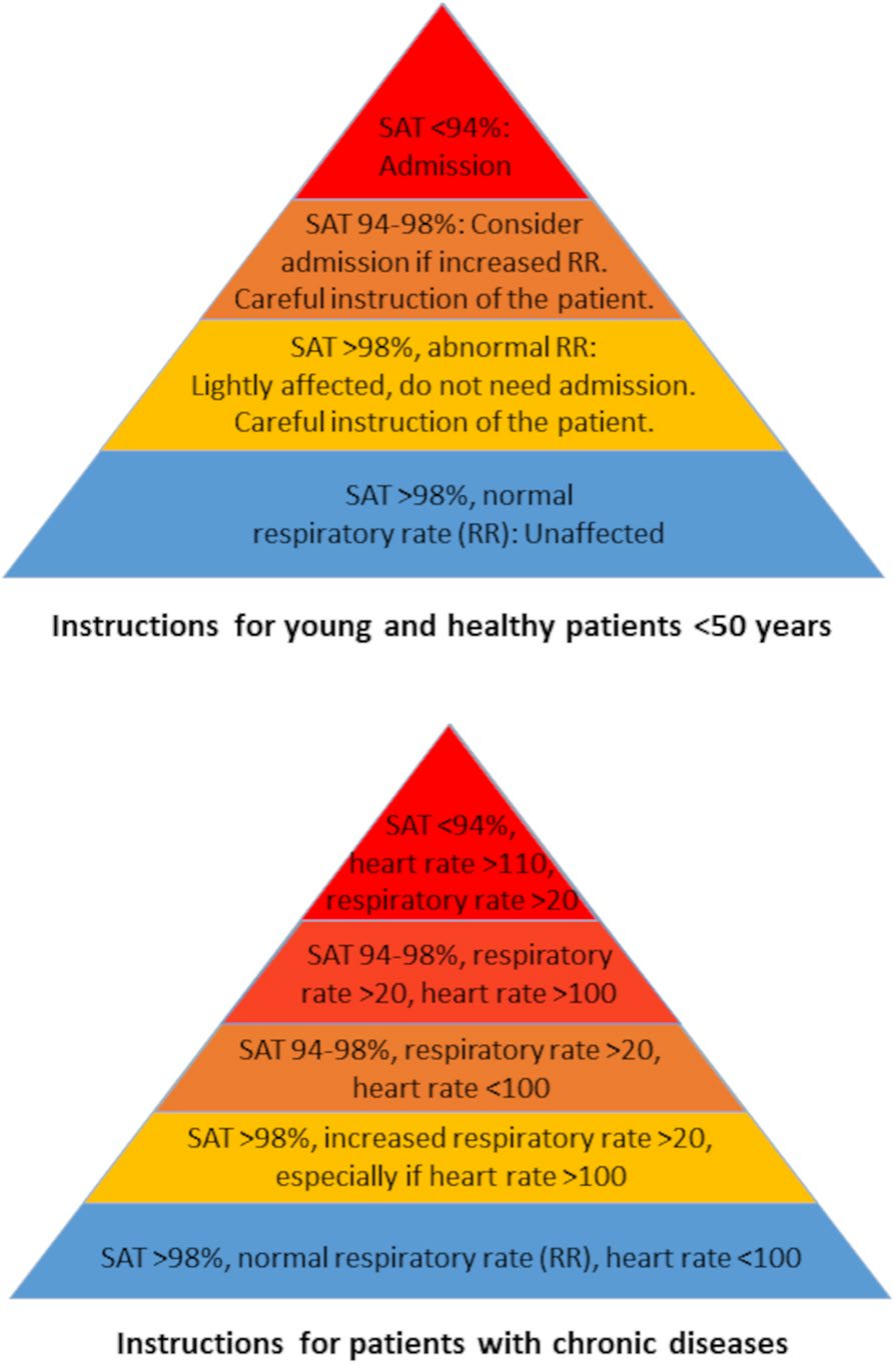
Vital sign limits according to the instruction manual. *SAT,* peripheral capillary oxygen saturation. *RR*, respiratory rate. The *COVID-19 instruction manual for paramedics* can be read in full length in Additional file 1

During the study period, 603 patients were referred to a paramedic’s assessment visit. We excluded 16 cases due to cancelled visits or missing outcome data, i.e. the prehospital ePMR did not contain a personal identification number necessary for the collection of follow-up data (Fig. 1). The final study population included 587 patients of which 449 patients (76.5%, 95% CI 72.8;79.9) were non-conveyed while 138 patients (23.5%, 95% CI 20.1;27.2) were directly conveyed to a hospital. More than half of the patients were aged 65 years or above (Table 1). The majority of the directly conveyed patients had no (CCI 0) or mild co-comorbidities (CCI 1–2), while CCI scores were missing in most of the non-conveyed patients. 431 conference calls were made, and with physician support, the paramedic decided on non-conveyance in 321 (74.5%) of those cases. Generally, more of the directly conveyed patients had vital signs outside the normal ranges compared to the non-conveyed patients (Fig. 2 and Table 2). Most of the included patients were fully capable of giving consent to the suggested plan and treatment (“yes” (69.5%), “no” (0.7%), “could not be obtained” (3.6%) and “missing information” (26.2%)). In most of the cases where the patient or caregiver was unable to consent to the plan, it was due to the patient not being fully conscious or having no language beforehand. In these cases, the seriousness of the situation prompted the paramedic to treat without consent.

**Table 1:** Baseline characteristics for patients suspected of COVID-19 whom paramedics assessed at home after referral from their GP or an out-of-hours GP

	Directly conveyed (*n *= 138)	Non-conveyed (*n* = 449)	Total (*N* = 587)
Response time^a^			
Median (IQR), minutes	25 (15–39)	28 (18–45)	27 (17–43)
Missing data, n (%)	4 (2.9)	2 (0.4)	6 (1.0)
Sex, n (%)			
Male	69 (50.0)	193 (43.0)	262 (44.6)
Missing data	0 (0.0)	10 (2.2)	10 (1.7)
Age, n (%)			
<20 years	2 (1.5)	5 (1.1)	7 (1.2)
20–65 years	33 (23.9)	133 (29.6)	166 (28.3)
65–90 years	88 (63.8)	261 (58.1)	349 (59.5)
>90 years	15 (10.9)	40 (8.9)	55 (9.4)
Missing data	0 (0.0)	10 (2.2)	10 (1.7)
Charlson Comorbidity Index (CCI), n (%)			
CCI 0	48 (34.8)	29 (6.5)	77 (13.1)
CCI 1–2	55 (39.9)	39 (8.7)	94 (16.0)
CCI≥3	23 (16.7)	15 (3.3)	38 (6.5)
Missing data	12 (8.7)	366 (81.5)	378 (64.4)
Conference call^b^			
Pulmonologist	36 (26.1)	64 (14.3)	100 (17.0)
Patient’s GP or OOH-GP	60 (43.5)	234 (52.1)	294 (50.1)
Prehospital physician	5 (3.6)	10 (2.2)	15 (2.6)
Infectious disease specialist	9 (6.5)	13 (2.9)	22 (3.7)
None	24 (17.4)	126 (28.1)	150 (25.6)
Missing data	4 (2.9)	2 (0.4)	6 (1.0)

*IQR,* Interquartile range. *(OOH)-GP*, Out-of-hours general practitioner

^a^ Time from dispatch of paramedic vehicle to arrival at the scene

^b^ If a paramedic had several conference calls during the visit, the physician who decided on (non)-conveyance is the one listed in the table

**Table 2:** Baseline vital signs for patients suspected of COVID-19 whom paramedics assessed at home after referral from their GP or an out-of-hours GP

	Directly conveyed (*n* =138)	Non-conveyed (*n*=449)	*p*-value*
Respiratory rate (RR), min^−1^			<0.01
RR 8–25	82 (59.4)	379 (84.4)	
RR 26–35	46 (33.3)	19 (4.2)	
RR>35 or<8	7 (5.1)	5 (1.1)	
Missing data, n (%)	3 (2.2)	46 (10.2)	
SpO_2_			<0.01
≥95%	46 (33.3)	248 (55.2)	
<95%	82 (59.4)	124 (27.6)	
Missing data	10 (7.2)	77 (17.1)	
Heart rate (HR), min^−1^			<0.01
HR 50–110	94 (68.1)	369 (82.2)	
HR<50	<5 (<3.6)	4 (0.9)	
HR>110	40 (29.0)	34 (7.6)	
Missing data	<5 (<3.6)	42 (9.4)	
Systolic blood pressure (SBP), mmHg			<0.01
SBP≥90	127 (92.0)	371 (82.6)	
SBP<90 mmHg	3 (2.2)	3 (0.7)	
Missing data	8 (5.8)	75 (16.7)	
Glasgow Coma Score (GCS)			<0.01
GCS=15	109 (79.0)	331 (73.7)	
GCS=14	11 (8.0)	6 (1.3)	
GCS≤13	5 (3.6)	5 (1.1)	
Missing data	13 (9.4)	107 (23.8)	
Temperature, °C			<0.01
35–38 °C	105 (76.1)	374 (83.3)	
>38 °C	18 (13.0)	17 (3.8)	
Missing data	15 (10.9)	58 (12.9)	

*IQR,* Interquartile range. *GP*, General practitioner. *SpO2*, Peripheral capillary oxygen saturation

^*^ Calculated by Chi-square or Fisher’s Exact test as appropriate

### Subsequent hospital visits and diagnoses

Fifty-nine of the 449 patients who initially stayed at home (13.1%, 95% CI 10.2;16.6) were referred to a hospital within 72 hours of the paramedic’s assessment visit. The majority of those patients arrived at a hospital after contact with their GP or an out-of-hours GP. Amongst the 449 non-conveyed patients, 13 (2.9%) had re-enquiries to the EMS followed by a hospital admission within 72 hours. There were 4 re-enquiries to the EMS that did not result in a hospital admission. Of all the 197 patients who visited a hospital, either directly after the paramedic’s assessment visit or within 72 hours of the visit, 49.2% received a COVID-19 diagnosis at some point during the hospital stay (either *Z038PA1: Observation due to reasonable suspicion of COVID-19 infection*, *B342A: COVID-19 infection without further specification* or *B972A: COVID-19 severe acute respiratory distress syndrome*). The remaining patients mainly had hospital diagnoses within the ICD-10 chapters of diseases in the respiratory (14.2%), circulatory (7.1%), digestive (3.0%) or genitourinary (2.5%) systems, or infections (4.6%).

### Short-term mortality

Overall mortality for the total study population was 8.5% (95% CI 6.5;11.1). Within 30 days of the visit, 40 of the 566 patients had died (7.1%, 95% CI 5.2;9.5). Among patients directly conveyed to a hospital, 30-day mortality was 11.1% (95% CI 6.9;17.9) as opposed to 5.8% (95% CI 4.0;8.5) among non-conveyed patients (unadjusted hazard ratio 1.4 [0.7;2.7], age- and sex-adjusted hazard ratio 1.3 [0.6;2.7]). Further ePMR review revealed that the majority of non-conveyed patients who died within 30 days either had ‘do-not-resuscitate’ orders, palliative care plans, severe comorbidities, age at or above 90 years or were nursing home residents (Table 3). Concomitant diseases among deceased patients included diabetes, metastatic cancer* (an asterisk (*) denotes’severe comorbidities’ if in an advanced stage), dementia*, schizophrenia, chronic obstructive pulmonary disease*, alcoholism, coronary artery disease, previous myocardial infarction, previous stroke, pulmonary fibrosis*, foot ulcers, heart failure*, atrial fibrillation, and hypertension. 30-day mortality was higher among hospitalised patients diagnosed with COVID-19 than among patients who were hospitalised for other reasons (13.8% (95% CI 8.3;23.0) versus 7.1% (95% CI 3.5;14.6)), however this difference was not significant.

**Table 3:** All-cause mortality for patients assessed by a paramedic at home after referral from their GP or an out-of-hours GP (*n*=566)

Days from visit	Directly conveyed (*n*=135)		Non-conveyed (*n*=431)	
Death within 3 days % [95% CI]	3.0	[1.1;7.8]	2.1	[1.1;4.0]
‘Expected deaths’ according to (a)^a^, n	<5		9	
Deaths without any of the circumstances mentioned in (a), n	<5		0	
Death within 7 days % [95% CI]	5.2	[2.5;10.7]	3.5	[2.1;5.7]
‘Expected deaths’ according to (a), n	5		15	
Deaths without any of the circumstances mentioned in (a), n	<5		0	
Death within 30 days % [95% CI]	11.1	[6.9;17.9]	5.8	[4.0;8.5]
‘Expected deaths’ according to (a), n	11		23	
Deaths without any of the circumstances mentioned in (a), n	<5		<5	

^a^(a) Either ‘do-not-resuscitate’ orders, palliative care plans, severe comorbidities, age≥90 years or nursing home residents

## Discussion

### Key results

The study concerns the initial two months of the COVID-19 pandemic when test capacity was restricted, and assessment clinics and treatment were mainly organised on a regional level. During the two months in which this prehospital arrangement was in place, three of four patients were non-conveyed and thus did not have to go to a COVID-19 assessment clinic or an emergency department. In one out of eight non-conveyance situations, the patient still went to a hospital within the following 72 hours of the paramedic’s assessment visit.

### Interpretation

In the two-month period, an average of five hospital visits a day were ‘prevented’ by assessing the patient at home. Five visits a day may not seem notable. On the other hand, if those patients had arrived at a hospital, they would have had to be located in isolated hospital wards with strict protocols for personal protective equipment and subsequent time- and resource-consuming decontamination procedures. Non-conveyance may also have been preferable to the patient in terms of avoiding transportation, reduced waiting time and reduced risk of a nosocomial COVID-19 infection.

The concept of non-conveyance is of great interest at the moment both among EMS and other stakeholders, patients and researchers [8–15]. The decision on non-conveyance is influenced by various factors both at organisational and patient level and not only the level of severity of the medical condition [10, 16]. Evaluation of safety of non-conveyance protocols is complex. Secondary contacts are widely used as an indirect outcome of safety [8, 17], yet alone it may be insufficient. It should preferably be supplemented by measures of morbidity or mortality. Our results for 30-day mortality are similar to Scottish EMS patients suspected of being COVID-19 positive [18]: 8.8% (UK) versus 11.1% (DK) among patients directly conveyed to hospital and 4.3% (UK) versus 5.8% (DK) among non-conveyed patients. In an unselected cohort of Danish patients who were attended by a medical emergency care unit with prehospital physicians and released on scene, mortality was 4.5% at 30 days [19]. It is plausible that the trend towards higher mortality for patients directly conveyed to hospital could be explained by the severity of the patients’ clinical condition. The mortality for non-conveyed patients was however slightly higher compared to the aforementioned studies. Nonetheless, all deaths but two in this group were regarded as ‘expected’ due to concomitant chronic diseases or advanced age.

### Strengths and limitations

The study is strengthened by minimal loss to follow-up since less than 1% of the study population had missing outcome data. Data on the deceased patients’ comorbidities were not systematically collected via a public health register but from the prehospital ePMR. Consequently, the patients could have had either undiagnosed comorbidities or severe comorbidities stated on their hospital record or their record at the GP’s office. For this reason, the study design limits an in-depth exploration of the cases of deceased patients. The aforementioned UK study reported a markedly higher 30-day mortality for laboratory confirmed COVID-19 cases (around 30%) [18]. Their study cohort included patients presenting with COVID-19 symptoms who had called the UK emergency number, 999, and thus is not directly comparable to our study population as we included patients who had called their GP or an out-of-hours GP. We did not collect test results from the throat swabs performed by the visiting paramedic and thus cannot conclude on the mortality of confirmed COVID-19 cases in the non-conveyance group. This may be seen as a limitation of the study. Yet, the PCR test result was not available to the visiting paramedic at the time of the decision. In this way, the study design reflects the real-life working conditions of the visiting paramedics. The complexity of visitation practices is also clearly reflected in our results: only half of the patients actually had a hospital diagnosis related to COVID-19, even though the inclusion criteria were ‘symptoms suspected of COVID-19’. Naturally, the non-conveyed patients who stayed at home were never formally diagnosed. A specific diagnosis is not a prerequisite for the decision of non-conveyance, as long as the patient is referred to the appropriate level of care.

### Future perspectives

In light of the current trend of emergency department crowding in several European countries, the role of community paramedics is an area under rapid development and extension in Denmark. Such newly implemented health care initiatives must be evaluated to ensure patient safety, preferably by using patient-centred outcomes. The use of paramedics as advanced visitation units has a potential for cost reductions in acute health care systems [11, 20]*.* With the gradual decline of the COVID-19 pandemic, the North Denmark EMS has carried on with a similar arrangement in a different setup. A patient can be referred to a paramedic’s assessment visit at home in any situation where it is not clear to the medical dispatcher or district nurse if the patient requires hospital care. Our results imply it is inadequate to evaluate non-conveyance protocols only by using rates of secondary contacts. We must attentively monitor new non-conveyance arrangements, and we must carefully review any cases of deceased patients as well as monitor adherence to standard operating procedures. This can be done by regular audits of both the prehospital and in hospital medical records.

## Conclusions

Three of four patients were non-conveyed following clinical assessment and testing for COVID-19 at home. The majority of the non-conveyed patients did not visit a hospital for the following three days after a paramedic’s assessment visit. The study implies that this newly established prehospital arrangement served as a kind of gatekeeper for the region’s hospitals with regard to patients suspected of COVID-19. However, one out of eight non-conveyed patients were subsequently referred to a hospital within three days of a paramedic’s assessment visit. This reflects the challenge of decision-making in the patient’s home when available information is limited to clinical presentation including a C-reactive protein test. The majority of the non-conveyed patients who died within 30 days had either ‘do-not-resuscitate’ orders, concomitant chronic diseases or advanced age, however not all of them. The study demonstrates that implementation of non-conveyance protocols should be accompanied by careful and regular evaluation to ensure patient safety.

## Supplementary Information

**Additional file 1.**

## Data Availability

The data that support the findings of this study are available from the North Denmark Region but restrictions apply to the availability of these data, which were used under license for the current study, and so are not publicly available. Data are however available from authors V.M.L.N. and T.A.L. upon reasonable request and with permission of the North Denmark Region.

## Abbreviations

CCI: Charlson co-morbidity index
COVID-19: Coronavirus disease 2019
EMS: Emergency medical services
ePMR: Electronic patient medical record
GP: General practitioner (of medicine)
ICD-10: International Classification of Diseases, Tenth Revision
Logis CAD: Logis (trade name) computer-aided dispatch
PCR: Polymerase chain reaction
