# Supplementary material for: Decision on non-conveyance of patients suspected of COVID-19 in a novel arrangement with assessment visits by paramedics at home
Source: BMC Emerg Med. 2023 May 26;23:56. doi: 10.1186/s12873-023-00826-6 (PMC10212731; doi:10.1186/s12873-023-00826-6)
Supplement: Supplementary file 1 — Additional file 1. [file 12873_2023_826_MOESM1_ESM.pdf]

Additional file 1 to Nielsen et al. *Decision on non-conveyance of patients suspected of COVID-19 in a novel arrangement with assessment visits by paramedics at home.* BMC Emerg Med.
DOI: 10.1186/s12873-023-00826-6

COVID-19 instruction manual for paramedics:

Young and healthy adults (<50 years):

Observe or measure temperature, oxygen saturation and respiratory rate:

Healthy patients are expected to have an oxygen saturation of 98-99%. Patients who must hyperventilate (>22/min) to maintain an oxygen saturation at this level can be considered acutely ill with moderate symptoms. They must be instructed to contact a physician in case of deterioration.

Patients with an oxygen saturation below 94% MUST be admitted to hospital. Patients with an oxygen saturation between 94% and 98% MAY be admitted to hospital depending on their general condition. If in doubt, consult a physician.

Be aware that patients could have secondary bacterial infections during the course of COVID-19. They may become septic at this point and present with an abnormal heart rate, blood pressure or an altered state of consciousness.

Patients with COVID-19 infections are not expected to have abnormal lung sounds on auscultation. Patients with bacterial pulmonary infections MAY have abnormal lung sounds on auscultation.

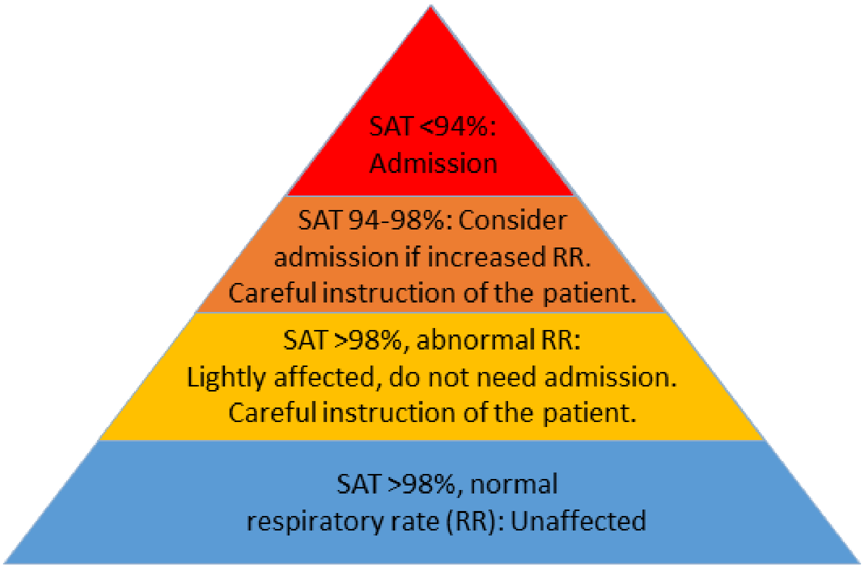

Instructions for young and healthy patients <50 years

Healthy adults (>50 years):

Basically as described above. However, you should pay a little more attention to the two middle categories of patients, especially the ones that have an oxygen saturation between 94 and 98%. You MAY choose to consult a physician in those cases. Keep in mind that patients in this age category could have undiagnosed chronic diseases. Patients with a respiratory rate >30 should be admitted to hospital. Be aware of an increased heart rate (>100). If in doubt, consult a physician.

Additional file 1 to Nielsen et al. *Decision on non-conveyance of patients suspected of COVID-19 in a novel arrangement with assessment visits by paramedics at home.* BMC Emerg Med.
DOI: 10.1186/s12873-023-00826-6

Patients with chronic diseases (all ages):

Pay special attention to patients who have heart diseases or kidney diseases, diabetes (especially type 2), chronic lung diseases, COPD, uncontrolled asthma, bronchiectasis and interstitial lung diseases as well as immunosuppression (cancer with active treatment, patients with organ transplants and patients with innate or acquired immune deficiencies).

Common to those patients are:

- They do not necessarily develop a fever.
- They may be fatigued more easily.
- Pay special attention to the oxygen saturation, respiratory rate and heart rate.
- In patients with heart disease, be aware that they may have been prescribed medications that could prevent the heart rate from increasing.
- Pay special attention to the increased risk of developing secondary bacterial infections in this patient category.

In all cases with patients with an oxygen saturation of 94-98%, you should confer with a physician. Patients with an oxygen saturation <94% should be admitted to hospital.

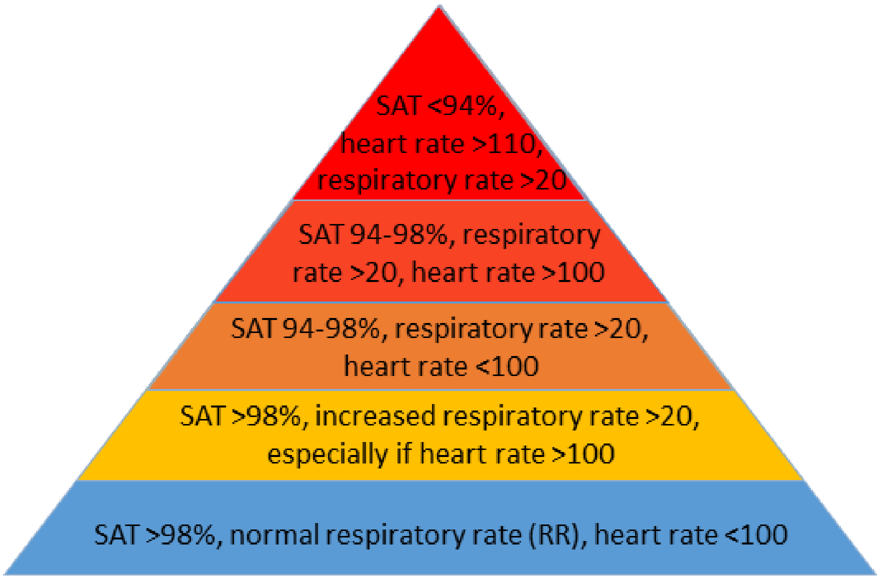

Instructions for patients with chronic diseases

UMW/March 2020
